# Dexmedetomidine and Ketamine Attenuated Neuropathic Pain Related Behaviors *via* STING Pathway to Induce ER-Phagy

**DOI:** 10.3389/fnsyn.2022.891803

**Published:** 2022-05-13

**Authors:** Yongda Liu, Shihui Kuai, Mengmeng Ding, Zhibin Wang, Limei Zhao, Ping Zhao

**Affiliations:** ^1^Department of Anesthesiology and Pain Management, Shengjing Hospital of China Medical University, Shenyang, China; ^2^Department of Pharmacy, Shengjing Hospital of China Medical University, Shenyang, China

**Keywords:** neuropathic pain, endoplasmic reticulum stress, autophagy, ketamine, dexmedetomidine, STING pathway

## Abstract

Our previous work indicated that ER-phagy level had altered in spinal nerve ligation (SNL) rats. In this study, we investigated whether dexmedetomidine or ketamine exhibits anti-anxiety or anti-nociceptive effects *via* modulation of the spinal STING/TBK pathway to alter ER-phagy in SNL rats. We evaluated the analgesic and anti-anxiety effects of ketamine and dexmedetomidine in SNL rats. 2’3’-cGAMP (a STING pathway agonist) was administrated to investigate whether enhanced spinal STING pathway activation could inhibit dexmedetomidine or ketamine treatment effects in SNL rats. Analgesic effects were assessed with the mechanical withdrawal threshold (MWT) and anti-anxiety effects were measured *via* an open field test (OFT). Protein expression levels were evaluated by immunoblotting. Distribution and cellular localization of Grp78 (ER stress marker) were evaluated by confocal immunofluorescence. SNL induced mechanical hypersensitivity and anxiety in rats; dexmedetomidine and ketamine both provided analgesia and anti-anxiety effects in SNL rats. Furthermore, the STING pathway was involved in the modulation of ER stress and ER-phagy in SNL rats and dexmedetomidine and ketamine alleviated ER stress by inhibiting STING pathway to enhance ER-phagy. Thus, both ketamine and dexmedetomidine provided anti-anxiety and anti-nociceptive effects by alleviating ER stress through the inhibition of the STING/TBK pathway to modulate spinal ER-phagy in SNL rats.

## Introduction

Neuropathic pain, which results from nervous system dysfunction caused by nervous system damage, can be a refractory disease (Costigan et al., 2009). Neuroinflammation contributes to neuropathic pain as a maladaptive mechanism, which originally promotes regeneration and healing following nerve damage (Skaper et al., 2018; Sommer et al., 2018; Chen et al., 2019). Furthermore, inflammation mediators could be promising biomarkers and treatments for neuropathic pain (Sommer et al., 2018).

Autophagy attenuates inflammatory signal transductions and removes aggregated inflammationsome components, suggesting autophagy provides a neuroprotective effect *via* inhibiting neuroinflammation (Cho et al., 2020). Autophagy has a protective effect, attenuating neuropathic pain *via* the restoration of cellular homeostasis in the spinal cord (Marinelli et al., 2014). Autophagy following ER stress primarily induced ER-phagy, which selectively degrades excess ER *via* autophagy processing (Senft and Ronai, 2015; Grumati et al., 2018; Loi et al., 2018; Sisinni et al., 2019). Thus, ER-phagy might be a promising treatment target for neuropathic pain. In this study, we examined glucose-regulated protein (Grp78) as an ER stress marker. The study demonstrated that STING (Stimulator of interferon genes) is essential to nociception (Donnelly et al., 2021). STING, an ER-resident protein related to neuroinflammation, has been identified as a novel mediator of crosstalk between ER stress and ER-phagy (Moretti et al., 2017; Gui et al., 2019; Yang et al., 2019; Paul et al., 2021). STING serves as an ER adaptor, being required under conditions of elevated ER stress (Petrasek et al., 2013; Moretti et al., 2017). STING/TBK (TANK binding kinase) pathway plays an essential role in ER stress. We evaluated autophagy processing of microtube-associated protein 1 light chain 3 (LC3, an autophagosome formation marker) and p62 (a marker of autophagic degradation) and evaluated formation levels of ER-phagy using FAM134b. FAM134b is an ER-resident receptor that binds LC3 and delivers ER to the lysosome for degradation.

Dexmedetomidine, a selective α2 adrenergic receptor (AR) agonist, provided analgesic potency during systemic administration (Yeo and Park, 2018; Liu et al., 2020b), low doses of dexmedetomidine also relieved tactile allodynia induced in a neuropathic pain model (Lin et al., 2018). Recent studies indicated that dexmedetomidine alleviated inflammation and protected the immune function in animals and patients (Wang et al., 2019; Mei et al., 2021).

Low doses of sedative ketamine, including doses at which it is used as an anti-depressant, could be a promising treatment for neuropathic pain (Orhurhu et al., 2019). Ketamine could provide neuroprotective effects and ameliorate neuroinflammation (Goncalves et al., 2021). Inhibition of spinal JNK activation contributed to the analgesic effects of ketamine in neuropathic pain (Mei et al., 2011).

Previous studies suggested that ketamine and dexmedetomidine could modulate the level of autophagy and ER stress (Chai et al., 2020; Li et al., 2020; Liu et al., 2020a; Sun et al., 2020; Yu et al., 2021). Our previous studies suggested that spinal ER-phagy was altered in the SNL-induced neuropathic pain model (Liu et al., 2017, 2019b). However, whether ketamine and dexmedetomidine could alter STING pathway to modulate ER-phagy and ER stress in SNL rats needs to be investigated. In this study, our aim was to investigate the effects of dexmedetomidine and ketamine administration on anxiety- and nociceptive-related behaviors and to investigate whether dexmedetomidine and ketamine alter spinal ER-phagy in SNL rats *via* modulation of the STING/TBK pathway.

## Materials and Methods

### Animals

**Figure 1:**
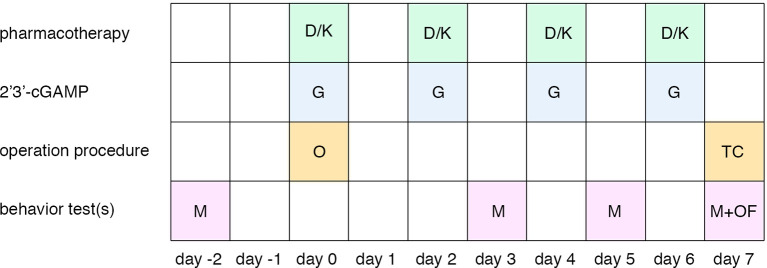
Timeline of the experimental procedure. Abbreviations: D, dexmedetomidine administration; K, ketamine administration; G, 2’3’-cGAMP administration; O, spinal nerve ligation operation; TC, tissue collection; M, mechanical withdrawal threshold; OF, open field test.

Male Sprague Dawley (SD) rats (180–230 g) were obtained from the Changsheng Biological Company and housed in the Shengjing Hospital Benxi experimental institution. This study was performed in accordance with China Medical University ethical guidelines for the use of laboratory animals and was approved by the Animal Ethics Care and Use Committee of China Medical University’s, Shengjing Hospital (Approval No. 2016PS013 K). All surgeries were performed under 10% chloral hydrate (0.4 ml/100 g) anesthesias. The timeline of experimental procedures is illustrated in Figure 1. Sham group: rats received sham SNL procedure; SNL group: rats received spinal nerve ligation; control group: rats received a sham procedure, a vehicle of dexmedetomidine/ketamine): rats received the SNL procedure, a vehicle of dexmedetomidine/ketamine, and vehicle of 2’3’-cGAMP; SNL + D group: rats received SNL, dexmedetomidine, and vehicle of 2’3’-cGAMP. SNL + D + G group: rats received SNL, dexmedetomidine, and 2’3’-cGAMP; SNL + K group: rats received SNL, ketamine, and vehicle of 2’3’-cGAMP. SNL + K + G group: rats received SNL, ketamine, and 2’3’-cGAMP. *N* = 6 per group.

### Spinal Nerve Ligation (SNL)

Spinal nerve ligation procedures were carried out as described previously (Kim and Chung, 1992). Rats were placed in the prone position under anesthesia. A 2 cm incision was made at left lumber 5, approximately 0.5 cm from the midline. The left L5 and L6 spinal nerves were separated and ligated tightly with a 4–0 silk suture; the distal end of the ligation was transected.

### Behavioral Assays

#### Mechanical Withdrawal Threshold (MWT)

To examine the mechanical hypersensitivity of rats, the mechanical withdrawal threshold (MWT) test was conducted using von Frey filaments (Stoelting Company, Wood Dale, IL, USA) as described previously (Zhou et al., 2014; Ge et al., 2018). Rats were habituated in a Plexiglas chamber for 30 min before conducting the MWT test. The positive withdrawal threshold of the hind paw was measured using the up and down procedure. For each MWT trial, the stimulation duration was approximately 5 s; the interval time was 5 min and the cut-off value was 15 g.

#### Open Field Test (OFT)

Anxiety and locomotor activity levels were evaluated with an open field test as reported previously (Kontinen et al., 1999). The open field arena consisted of an aluminum plate base (100 cm × 100 cm) surrounded by walls 45 cm high. The interior was painted black. The open field arena was equipped with infrared detectors and analyzed *via* Noldus software. Rats were put in the open field for 10 min. The total distance traveled, the proportion of time spent in the center zone, travel trace, and heat map were recorded. The field was cleaned with 75% ethanol after each trial.

### Drug Delivery

Intrathecal injections of 10 μg 2’3’-cGAMP (STING agonist, Cat. No. tlrl-nacga23, InvivoGen, USA) were performed *via* a 10 μl Hamilton microsyringe during the SNL procedure and repeated on post-operative days 2, 4, and 6. Single intrathecal injections were administrated *via* percutaneous lumbar puncture between the 5th or 6th intervertebral space. A rapid tail flick indicated that the microsyringe had penetrated the dura mater. The injection speed was approximately 0.5 s/μl and the microsyringe was maintained immobile for 1 min after the injection.

Ketamine (20 mg/kg, Cat.#: 1709291, Fujian Gutian Pharmaceutical Co., Ltd. China) was intraperitoneally (i.p.) injected at 6 h prior to surgery and again on postoperative days 2, 4, and 6. Dexmedetomidine (20 μg/kg, Cat. No. 181017BP, Hengrui Pharmaceutical Co., Ltd. China) was intraperitoneally (i.p.) injected 6 h prior to surgery and again on postoperative days 2, 4, and 6. The control (con) group received a sham operation along with intraperitoneal and intrathecal vehicle injections.

### Western Blot

On postoperative day 7, SD rats were deeply anesthetized and sacrificed. Spinal cords between L4 and L6 were rapidly dissected and frozen at −80°C. Then, tissues were homogenized in RIPA buffer (p0013B, Beyotime, China) and phosphorylation inhibitors (1:100, Solarbio, China) for 30 min on ice, followed by centrifugation at 14,000 rpm for 40 min at 4°C. The supernatant fraction was collected; the resulting lysate was mixed with loading buffer (Beyotime, China) and separated with a 12% SDS/PAGE gel and transferred to a PVDF membrane (GE, USA). Each primary antibody was incubated with the membrane at 4°C overnight (<12 h)after blocking with 5% BSA with TBST (0.1% Tween 20 in Tris-buffered saline) for 1 h at room temperature. Blots were incubated with HRP-conjugated second antibodies for 1.5 h at room temperature. After three washes with TBST, a signal was detected using an ECL Plus kit (Tanon, China bands), then visualized using a chemiluminescence imaging system (GE, USA; c300, Azure Biosystems, USA). Band intensities were quantified with Image J software (NIH, USA).

The following antibodies were used in this study: rabbit anti-Grp78 (1:2,000, Abcam, USA), rabbit anti-LC3 (1:1,000, CST, USA), rabbit anti-p62 (1:2,000, CST, USA), rabbit anti-FAM134B (1:1,000, Abcam, USA), rabbit anti-p-STING (1:1,000, CST, USA), rabbit anti-STING (1:1,000, CST, USA), rabbit anti-p-TBK (1:1,000, CST, USA), rabbit anti-TBK (1:1,000, CST, USA), mouse anti-GAPDH (1:8,000, Solarbio, China), and goat anti-rabbit/goat anti-mouse IgG horseradish peroxidase (1:5,000, Beyotime, China).

### Immunofluorescence Staining

Rats were deeply anesthetized, and underwent transcardial perfusion with a 0.9% NaCl solution, followed by cold 4% Paraformaldehyde in 0.1 M PBS. L5 spinal cords were removed, fixed in fixative solution for 24 h, then dehydrated with 30% sucrose in dd H_2_O at 4°C for 24 h. Brains were embedded with optimal cutting temperature (OCT, SAKURA, USA) compound. Embedded L5 tissue was sectioned coronally at 10 μm thickness using a cryostat. For confocal immunostaining, sections were incubated with anti-NeuN (neuronal marker, 1:200, MAB377, Millipore, USA)/anti-GFAP (glial cell marker, 1:200, Abcam, USA) and anti-Grp78 (ER stress marker, 1:200, Abcam, USA)/anti-Grp78 (1:200, Abcam, USA). Cell nuclei were counterstained with DAPI (Beyotime, China) for 5 min.

### Statistical Analysis

Data are expressed as the mean ± standard error of the mean (SEM). Analysis was conducted using IBM SPSS Statistics 22 software (SPSS Inc., Armonk, New York, USA). Western blot and open field test results were analyzed by one-way analysis of variance (ANOVA) following *post hoc* multiple comparisons; MWT data were analyzed by two-way analysis of variance (ANOVA) following *post hoc* multiple comparisons (effectors: time and surgery). *P* values < 0.05 were considered significant.

## Results

### Spinal Nerve Ligation Led to Severe Mechanical Hypersensitivity, Anxiety, and STING/TBK Activation

**Figure 2:**
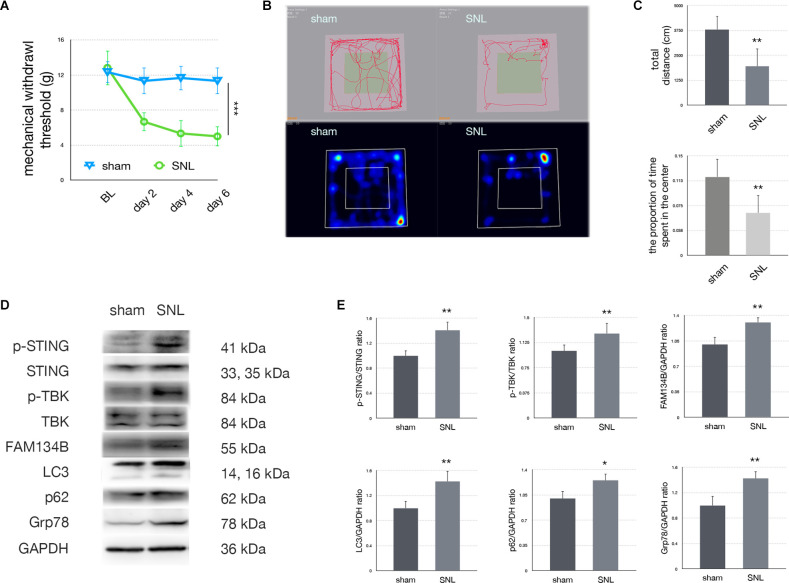
Spinal nerve ligation activated the STING/TBK pathway. **(A)**Mechanical withdrawal threshold in sham vs. SNL groups (two-wayANOVA, *P* < 0.001). **(B)** Trace map and heat map ofthe open field test in sham vs. SNL groups. **(C)**Quantification of the open field test (one-way ANOVA, total distance:*F* = 16.781, *P* = 0.002; proportion of time spent in the center: *F* = 12.512, *P* = 0.005). **(D)** Western blotting in sham vs. SNL groups. **(E)** Quantification of immunoblotting in sham vs. SNL groups (one-way ANOVA, p-STING/STING: *F* = 16.75, *P* = 0.002; p-TBK/TBK: *F* = 10.786, *P* = 0.008; FAM134b/GAPDH: *F* = 19.265, *P* = 0.001; LC3/GAPDH: *F* = 10.756, *P* = 0.008; p62/GAPDH: *F* = 8.957, *P* = 0.014; Grp78/GAPDH: *F* = 10.897, *P* = 0.008). *N* = 6 rats per group, *P < 0.05 compared with the sham group; **P < 0.01 compared with sham group; ***P < 0.001 compared with sham group. Abbreviations: SNL, spinal nerve ligation; sham, sham operation of spinal nerve ligation; BL, baseline.

A significant decrease in MWT was observed on postoperative days 2, 4, and 6. Since anxiety is a frequent co-morbidity of neuropathic pain, anxiety levels were also evaluated with OFT. Our data demonstrated that the total distance traveled and the proportion of time spent in the center zone significantly decreased in comparison to the sham group. Representative heat maps and travel traces are shown in Figure 2. Expressions of related proteins were evaluated using Western blotting. Upregulation of LC3, FAM134b, Grp78, and p62 confirmed that SNL induced ER-phagy impairment and ER stress. Furthermore, p-STING/STING and p-TBK/TBK ratios were increased in the SNL group compared with the sham group, indicating that the STING/TBK pathway was activated in rats undergoing SNL.

### Distribution and Cellular Localization of Grp78 in SNL Rats

**Figure 3:**
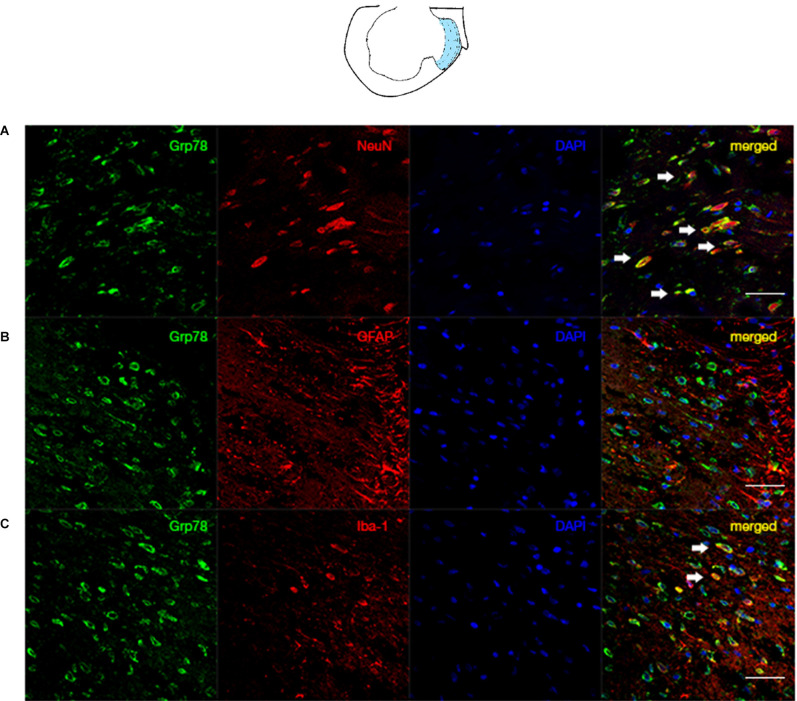
Co-localization of Grp78 with NeuN/GFAP/Iba in the ipsilateral spinal dorsal horn of SNL rats. The laminae I-III was illustrated by the blue zone in the ipsilateralL5 atlas. **(A)** Confocal immunofluorescence of Grp78 and NeuN (neuron marker) in laminae I-III of SNL rats at L5 level. **(B)** Confocal immunofluorescence of Grp78 and GFAP (astrocyte marker) in laminae I-III of SNL rats at L5 level. **(C)** Confocal immunofluorescence of Grp78 and Iba (microglia marker) in laminae I-III of SNL rats at L5 level. *N* = 6 rats per group. Scale bar = 50 μm.

Our data demonstrated that the ER stress marker, Grp78, was mainly expressed in neurons of laminae I-III (Figure 3). Our data suggest that the STING/TBK pathway is activated and that ER stress markers are expressed mainly in neurons following SNL.

### Effects on Pain Behavior Test Following Dexmedetomidine or Ketamine Administration

**Figure 4:**
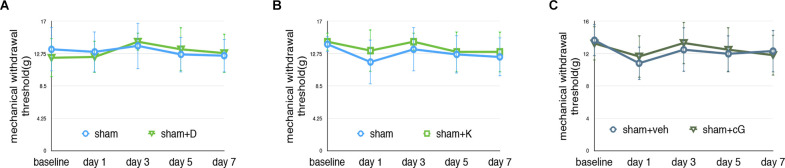
Effectson pain behavior test following dexmedetomidine or ketamine administration. **(A)** MWT of sham and sham+D groups, sham: sham of SNL groups; sham+D: sham+dexmedetomidine administration group. **(B)** MWT of sham and sham-K groups, sham: sham of SNL groups; sham-K: sham+ketamine administration group. There were no significant changes in sham+D/sham+K compared to the sham group. **(C)** MWT of the sham and sham+cG group. There were no significant changes between the sham and sham+cG group (*N* = 4, two-way ANOVA, *P* > 0.05).

As shown in Figure 4; our data confirmed that dexmedetomidine or ketamine did not change the MWT test of sham rats. In this study, we administrated 2’3’-cGAMP to elevate the activity of the STING pathway, we administrated 2’3’-cGAMP (5 μl) in sham rats. Our data suggested that there were no significant changes in sham+cG compared with the sham group (*N* = 4, two-way ANOVA, *P* > 0.05).

### Dexmedetomidine Neutralized SNL Induced Nociception, Anxiety, and STING/TBK Pathway Activation

**Figure 5:**
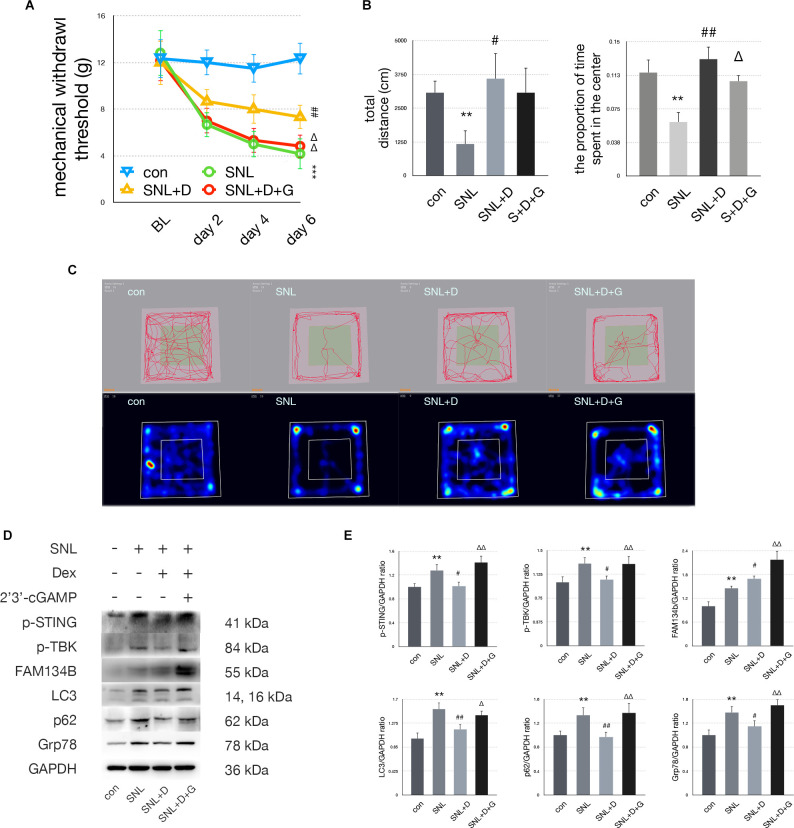
Dexmedetomidineprovided anti-anxiety effects by inhibiting the STING/TBKpathway. **(A)** MWT tests of control, SNL, SNL+D, and SNL+D+G groups (two-way ANOVA). **(B)** Quantification of open field of control, SNL, SNL+D, and SNL+D+G groups (one-way ANOVA, total distance: *F* = 13.260, *P* = 0.003; proportion of time spent in the center: *F* = 16.520, *P* = 0.006). **(C)** Trace map and heat map of control, SNL, SNL+D, and SNL+D+G groups. **(D)** Western blot of control, SNL, SNL+D, and SNL+D+G groups. **(E)** Western blot quantification of control, SNL, SNL+D, and SNL+D+G groups (one-way ANOVA, p-STING/GAPDH: *F* = 6.092, *P* = 0.006; p-TBK/GAPDH: *F* = 3.709, *P* = 0.034; FAM134b/GAPDH: *F* = 13.979, *P* = 0.007; LC3/GAPDH: *F* = 9.265, *P* = 0.001; p62/GAPDH: *F* = 4.268, *P* = 0.022; Grp78/GAPDH: *F* = 8.613, *P* = 0.001). *N* = 6 rats per group, *P < 0.05 compared with control; **P < 0.01 compared with control group;***P < 0.001 compared with control group;^#^*P* < 0.05 compared with SNL;^##^*P* < 0.01 compared with SNL group;^Δ^*P* < 0.05 compared with SNL+D;^ΔΔ^*P* < 0.01 compared with SNL+D. Abbreviations: BL, baseline; Dex, dexmedetomidine; control group, rats received a sham operation and vehicle; SNL group, rats received the SNL procedure and vehicle; SNL+D group, rats received SNL, dexmedetomidine, and vehicle; SNL+D+G group, rats received SNL, dexmedetomidine, and 2’3’-cGAMP.

In the SNL+D group, MWT was increased on postoperative days 4 and 6 compared with the SNL group (Figure 5A). Dexmedetomidine injection significantly increased the proportion of time rats spent in the center zone compared with the SNL group (Figure 5B). p-STING and p-TBK significantly decreased, indicating that the STING/TBK pathway was downregulated following dexmedetomidine administration. Upregulation of FAM134b and downregulation of LC3, p62, and Grp78 suggests ER-phagy was increased while ER stress was attenuated. Moreover, 2’3’-cGAMP administration reversed the analgesic and anti-anxiety effects induced by dexmedetomidine administration (Figures 5A,B). In the SNL+D+G group, p-STING, p-TBK, FAM134b, LC3, p62, and Grp78 were increased as compared with the SNL+D group, indicating that 2’3’-cGAMP administration induced ER-phagy impairment and ER stress by activating the STING/TBK pathway (Figure 5D). Representative heat maps and travel traces of the control, SNL, SNL+D, and SNL+D+G groups are shown in Figure 5C.

### Ketamine Neutralized SNL Induced Nociception, Anxiety, and STING/TBK Pathway Activation

**Figure 6:**
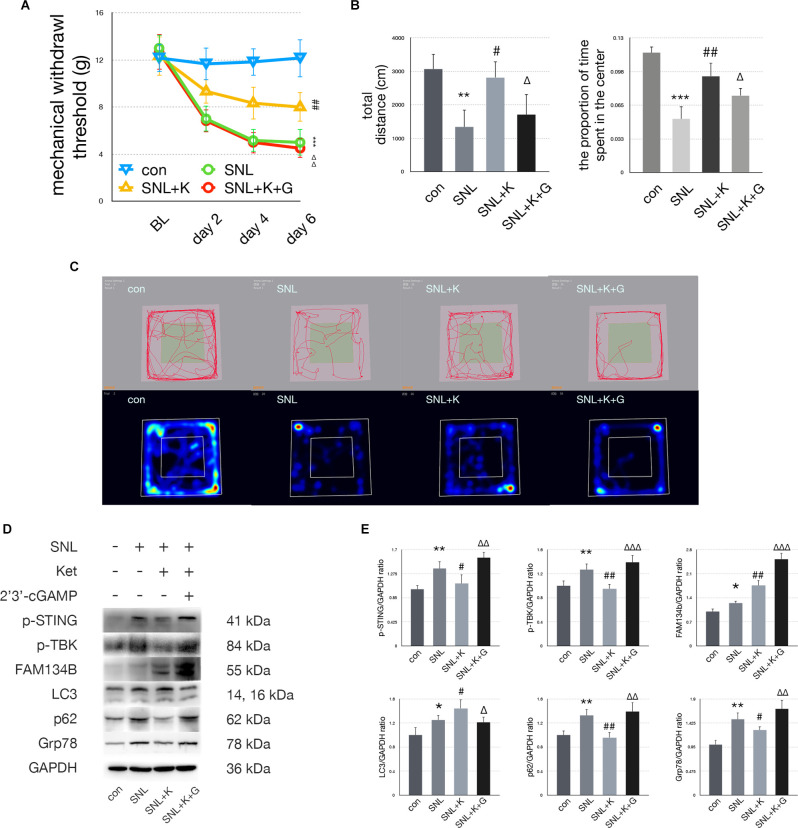
Ketamineprovided anti-anxiety effects by inhibiting the STING/TBKpathway. **(A)** MWT tests of control, SNL, SNL+K, and SNL+K+Ggroups (two-way ANOVA). **(B)** Quantification of open field ofcontrol, SNL, SNL + K, and SNL + K + G groups (one-way ANOVA, totaldistance: *F* = 16.034, *P* = 0.002; proportion of time spent in the center: *F* = 11.637, *P* = 0.005). **(C)** Trace map and heat map of control, SNL, SNL + K, and SNL + K + G groups. **(D)** Western blot of control, SNL, SNL + K, and SNL + K + G groups. **(E)** Western blot quantification of control, SNL, SNL + K, and SNL + K + G groups (one-way ANOVA, p-STING/GAPDH: *F* = 10.419, *P* = 0.001; p-TBK/GAPDH: *F* = 12.993, *P* = 0.003; FAM134b/GAPDH: *F* = 16.606, *P* = 0.005; LC3/GAPDH: *F* = 11.014, *P* = 0.005; p62/GAPDH: *F* = 4.047, *P* = 0.026; Grp78/GAPDH: *F* = 7.520, *P* = 0.002). *N* = 6 rats per group, *P < 0.05 compared with control; **P < 0.01 compared with control; ***P < 0.001 compared with control; ^#^*P* < 0.05 compared with SNL; ^##^*P* < 0.01 compared with SNL; ^Δ^*P* < 0.05 compared with SNL+K; ^ΔΔ^*P* < 0.01 compared with SNL+K; ^ΔΔΔ^*P* < 0.001 compared with SNL+K. Abbreviations: BL, baseline; Ket, ketamine; control group, rats received a sham operation and vehicle; SNL group, rats received the SNL procedure andvehicle; SNL+K group, rats received SNL, ketamine, and vehicle; SNL+K+G group, rats received SNL, ketamine, and 2’3’-cGAMP.

In the SNL+K group, MWT was increased on postoperative days 2, 4, and 6 compared with the SNL+V group. Ketamine injection significantly increased the total distance traveled and the proportion of time spent in the center zone as compared with the SNL group. Representative heat maps and travel traces are shown in Figure 6. Ketamine administration decreased the expression of p-STING, p-TBK, p62, and Grp78 and increased the expression of FAM134b and LC3 in comparison with the SNL group. These data suggest that ketamine ameliorated ER stress by increasing ER-phagy by inhibiting the STING/TBK pathway. Furthermore, 2’3’-cGAMP neutralized the analgesic and anti-anxiety effects induced following ketamine administration as assayed using MWT and OFT. Moreover, in the SNL+K+G group, the protein expression of p-STING, p-TBK, FAM134b, p62, and Grp78 was increased while the expression of LC3 was decreased as compared with the SNL+K group. Data revealed that 2’3’-cGAMP activated the STING/TBK pathway and inhibited ER-phagy, which enhanced ER stress.

## Discussion

Dexmedetomidine and ketamine have been previously reported to induce analgesic and anxiolytic effects when given as premedication before general anesthesia (Oriby, 2019). Dexmedetomidine, a highly selective α2 agonist, provides analgesic benefit *via* enhancing norepinephrine (NE) concentration in the spinal cord of neuropathic pain patients (Yang et al., 2018; Qian et al., 2019). Dexmedetomidine administration resulted in anti-allodynic and anxiolytic effects in animal neuropathic pain models (Liang et al., 2017; Fang et al., 2019; Qian et al., 2019), suggesting that dexmedetomidine might be a promising pharmacotherapy for neuropathic pain.

Prior studies have focused on the neurotoxicity of high-dose ketamine administration and long-term ketamine abuse (Li et al., 2019; Meng et al., 2020). However, ketamine is promising if used as a proper rescue pharmacotherapy or as an adjuvant medication for neuropathic pain (Orhurhu et al., 2019). Sub-anesthetic dosing of ketamine provided clinical and experimental benefits for chronic pain and depression (Humo et al., 2020; Yang et al., 2020). Therefore, it is essential to investigate the mechanism and efficacy of ketamine in a neuropathic pain model.

Administrations of dexmedetomidine and ketamine have both been shown to reverse central sensitization induced by neuropathic pain (Orhurhu et al., 2019; Xun and Zheng, 2020). However, little is known about the underlying molecular mechanisms by which dexmedetomidine and ketamine attenuate neuropathic pain-induced allodynia and anxiety.

Our data demonstrated that dexmedetomidine and ketamine reversed SNL-induced allodynia and anxiety significantly. As shown in Figures 5 and 6, mechanical allodynia was attenuated in the SNL+D and SNL+K groups as compared with the SNL group, respectively. Dexmedetomidine and ketamine administration also induced anxiolytic effects as measured *via* OFT. To be specific, dexmedetomidine increased the proportion of time rats spent in the center of the field. Similarly, ketamine increased both the total distance traveled and the proportion of time spent in the center of the field in SNL rats. Our data suggest that ketamine could both improve the performance of locomotion as well as ameliorate anxiety behaviors. In addition, dexmedetomidine and ketamine both provided a satisfactory analgesic effect in the SNL-induced neuropathic pain rat model.

To determine whether ER-phagy was modulated following dexmedetomidine and ketamine administration in SNL rats, the expression of FAM134b, LC3, and p62 were measured with immunoblotting. ER-phagy includes induction, formation, and degradation. The next step is to select the cargo that is to be removed or broken down. We measured the level of cargo selection with FAM134b, the FAM134b increase indicates more ER (endoplasmic reticulum) were selected as cargo. The membrane expands to completely enclose the cargo. This step gives rise to autophagosomes. In this study, we use LC3 to investigate the level of autophagosomes. LC3 elevating suggested autophagosomes level increased. Then, autophagosomes fuse with lysosomes for the degradation of cargo. In this study, we used p62 to evaluate the level of degradation. p62 decrease indicated the degradation step increased. Interestingly, our data revealed that ER-phagy processing was modulated differently by dexmedetomidine and ketamine administration. ER-phagy levels were altered in different ways following dexmedetomidine or ketamine treatment. Our data suggested that LC3 was significantly increased in parallel with FAM134b increased and p62 decreased in SNL+K group compared with the SNL group, suggesting that ketamine administration increased both the induction and degradation processing of ER-phagy in the spinal of SNL rats. On the other hand, dexmedetomidine administration induced LC3 decreased, p62 decreased and Fam134b increased suggesting that the degradation of ER-phagy process of ER-phagy was significantly upregulated. Our data suggested that both ketamine and dexmedetomidine increase the level of ER-phagy and attenuated ER-phagy impairment SNL induced. Ketamine enhanced ER-phagy formation and degradation, while dexmedetomidine enhanced ER-phagy degradation.

Our previous study suggested that dexmedetomidine administration could decrease the expression of Grp78, LC3, and p62 while increasing the expression of FAM134 bas compared with SNL rats (Liu et al., 2020b). We confirmed this result in this study. The level of ER binding to autophagosomes and ER degradation *via* ER-phagy was increased; however, autophagosome levels were decreased following dexmedetomidine administration in the spinal cord of SNL rats. The decrease in autophagosomes might have resulted from an imbalance in the formation and effective degradation of them. Ketamine treatment resulted in significant increases in FAM134b and LC3 while p62 significantly decreased, suggesting spinal ER-phagy formation and degradation significantly increased. Our data suggest that more ER was selectively removed by autophagosomes and degradation *via* ER-phagy processing. Dexmedetomidine increased degradation *via* ER-phagy, while ketamine induced both the formation and degradation of autophagosomes and increased the uptake of ER by the autophagosome. Regardless, both dexmedetomidine and ketamine administration led to enhanced ER-phagy.

Recently, several studies demonstrated that the STING pathway had tremendous potential for immunotherapy (Chon et al., 2019; Su et al., 2019; Catalano et al., 2020). Its conformational changes and phosphorylation result in its translocation from the ER to distinct perinuclear endosomes near the Golgi (Bai and Liu, 2019; Chon et al., 2019; Yang et al., 2019). STING phosphorylation induces phosphorylation of TBK1 and subsequently leads to the phosphorylation of interferon regulatory factor 3 (IRF3; Mathur et al., 2017; Fermaintt et al., 2019). However, whether the STING pathway is involved in the neuropathic pain process and whether STING can regulate ER-phagy remains unknown. In this study, our data indicate that the STING/TBK pathway was activated in the spinal cord of a rat neuropathic pain model system.

Activated STING signaling triggers ER stress (Wu et al., 2019). The STING-containing endoplasmic reticulum-Golgi intermediate compartment (ERGIC)was shown to serve as a membrane source for the lipidation of LC3 (Dong et al., 2015; Yang et al., 2019; Larabi et al., 2020). STING directly interacted with LC3 and regulated autophagy (Gui et al., 2019). Thus, STING modulates autophagy, an essential mechanism to maintain ER homeostasis following ER stress, *via* localizing to autophagosomes (Senft and Ronai, 2015; Grumati et al., 2018; Song et al., 2018; Wilkinson, 2019). Specifically, studies have suggested that altering the STING pathway drives ER-phagy to resolve ER stress (Moretti et al., 2017; Kary, 2018; Moretti and Blander, 2018).

In this study, we determined whether the STING pathway was activated in the spinal cord of rats with SNL-induced neuropathic pain following dexmedetomidine or ketamine injection. Our data reveal that dexmedetomidine and ketamine both upregulated ER-phagy by inhibiting the STING/TBK pathway. Moreover, 2’3’-cGAMP administration increased LC3 expression in the SNL + D + G group to a greater extent than in the SNL + D group, indicating that ER-phagy degradation level was decreased following 2’3’-cGAMP injection. On the other hand, 2’3’-cGAMP decreased LC3 expression in the SNL + K + G group as compared with the SNL + K group, suggesting STING activation led to ER-phagy induction decreased. In this condition, cGAMP elevated the excessive activation of the STING pathway and neutralized the anti-nociceptive and anxiolytic effects of ketamine and dexmedetomidine *via* altering different steps of ER-phagy.

We investigated the distribution and cellular localization of spinal ER stress using immunofluorescence. Grp78 was primarily expressed in neurons and microglial cells; these data indicate that SNL induces spinal ER stress in neurons by activating the STING pathway in the spinal cord. Studies indicated that STING is an essential immune response and nociception in neurons (Flood et al., 2019; Gui et al., 2019; Liu et al., 2019a; McLaughlin et al., 2020; Donnelly et al., 2021). Studies suggested that STING contributes to ER stress and several diseases (Smith, 2020; Zhang et al., 2020; Li et al., 2021). Our data suggested that excess and prolonged elevated p-STING neutralized the ketamine-induced benefits in SNL rats. Ji’s team investigated STING inducer attenuated menthol gel-induced cold allodynia in rhesus macaques, suggesting STING agonist (ADU-S100) induced analgesia in non-human primates. While our data suggested that RU-521, cGAS/STING pathway inhibitor, ameliorated SNL-induced hypersensitivity and depression. Ji’s study elucidated that administration of 2’3’-cGAMP (5 μl) provided an antinociceptive effect in acute pain models (Costigan et al., 2009), while our data suggested that repeated injecting 2’3’-cGAMP to excessively activate p-STING could deteriorate pain behavior in chronic pain SNL rats. The pain-related test in Ji’s team mainly collected behavior data the hours after injection; our data collected nociceptive- and anxiety-related behaviors days and weeks after surgery. Furthermore, STING might provide opposite effects in cancer therapies (Chon et al., 2019; Flood et al., 2019; Motwani et al., 2019; Wang et al., 2021). These data might provide opposite outcomes, it seems a paradox, but it revealed that STING is critical in human health and diseases. In addition, does and time effect relation could affect the outcomes of modulating STING. Still, further study could focus on neuron and microglial cells to investigate the mechanism underlying the STING pathway ER-phagy and ER stress in neuropathic pain condition.

However, this study failed to demonstrate whether dexmedetomidine and ketamine provide dose-dependent analgesic and anti-anxiety effects in SNL rats. Furthermore, the STING pathway might be involved in the immune response or crosstalk between ER-phagy and the immune response in neurons or glial cells; this area requires further investigation. Our data indicated that ketamine and dexmedetomidine might provide anti-nociceptive and anti-anxiety effects *via* neuroinflammation related STING pathway in SNL rats. However, our study failed to point out the domain dexmedetomidine and ketamine bind with STING or ER.

In this study, our data suggested that dexmedetomidine and ketamine provide analgesic and anti-anxiety effects on SNL rats. The STING/TBK signaling pathway was activated in SNL rats. Both dexmedetomidine and ketamine can increase ER-phagy *via* inhibiting the STING pathway, thus attenuating ER stress in SNL rats, though dexmedetomidine and ketamine increased ER-phagy in the spinal cord of SNL rats differently. Furthermore, we would like to discuss the roles of STING and ER-phagy in the pain matrix of a neuropathic pain model and investigate the behaviors and molecular changes in further study.

## Data Availability Statement

The raw data supporting the conclusions of this article will be made available by the authors, without undue reservation.

## Ethics Statement

The animal study was reviewed and approved by Animal Ethics Care and Use Committee of China Medical University, Shengjing Hospital.

## Author Contributions

YL designed the study and wrote the manuscript. SK, MD, and ZW analyzed data and revised the manuscript. YL and SK performed the research. PZ and LZ supervised the study. All authors contributed to the article and approved the submitted version.

## Conflict of Interest

The authors declare that the research was conducted in the absence of any commercial or financial relationships that could be construed as a potential conflict of interest.

## Publisher’s Note

All claims expressed in this article are solely those of the authors and do not necessarily represent those of their affiliated organizations, or those of the publisher, the editors and the reviewers. Any product that may be evaluated in this article, or claim that may be made by its manufacturer, is not guaranteed or endorsed by the publisher.
